# Study on Microstructure and Properties of K-TIG Welded Joint of 95 mm Ti-6Al-4V Thick Plate

**DOI:** 10.3390/ma18163848

**Published:** 2025-08-16

**Authors:** Yinqing Gong, Songxiao Hui, Yang Yu, Zhihao Zhang, Xiongyue Ye, Wenjun Ye, Zhongliang Wang

**Affiliations:** 1 State Key Laboratory of Nonferrous Structural Materials, China GRINM Group Co., Ltd., Beijing 100088, China; gongyinqing@163.com (Y.G.); yuyang@grinm.com (Y.Y.); yewenjun@grinm.com (W.Y.); 2 Key Laboratory for Advanced Materials Processing (MOE), Institute for Advanced Materials and Technology, University of Science and Technology Beijing, Beijing 100083, China; ntzzh2279@163.com; 3 Beijing Laboratory of Metallic Materials and Processing for Modern Transportation, Institute for Advanced Materials and Technology, University of Science and Technology Beijing, Beijing 100083, China; 4 GRIMAT Engineering Institute Co., Ltd., Beijing 101407, China; 5 General Research Institute for Nonferrous Metals, Beijing 100088, China; 6 GRINM (Guangdong) Institute for Advanced Materials and Technology, Foshan 528051, China; 7 Guangdong Foreweld Co., Ltd., Guangzhou 510300, China; 2020282080099@whu.edu.cn

**Keywords:** Ti-6Al-4V thick plate, K-TIG welding, phase composition, texture evolution, mechanical properties

## Abstract

This study investigates the application of the Keyhole–Tungsten Inert Gas Welding (K-TIG) hot-wire filling welding technique with mechanical arc oscillation to weld a 95 mm-thick Ti-6Al-4V titanium alloy plate. The root layer thickness achieved with this technique reaches up to 17 mm, with an average filling thickness of 2.5 mm. The weld bead displays a smooth, shiny appearance, and no significant welding defects are observed in the cross-section of the welded joint. Experimental results show that the welded joint consists of the α phase in different forms, as well as fine α+β microstructures. Compared to the base material, both the weld metal and the heat-affected zone exhibit a lower crystallographic texture strength, with more complex texture types. The impact toughness of the welded joint is excellent, with no significant weaknesses. The impact toughness of the weld metal significantly surpasses that of both the base material and the heat-affected zone. The engagement strengthening effect induced by high-current filling plays a crucial role in enhancing the impact toughness of the weld metal.

## 1. Introduction

Titanium and its alloys are prized for their high strength-to-weight ratio, excellent corrosion resistance, and high stiffness-to-weight ratio, making them essential structural materials in advanced marine engineering applications. These materials are widely utilized in offshore oil and gas extraction, manned submersibles, ships, and other critical fields [1,2,3]. In recent years, several major marine engineering equipment models under development have adopted a “full titanium construction” approach for key components, driving the extensive use of thick titanium alloy plates. Consequently, developing an efficient, cost-effective, and high-performance welding technique for titanium alloy thick plates is vital for enhancing the use of titanium alloy structural components.

High-energy beam welding methods, such as electron beam, laser, and plasma welding, offer high welding efficiency, low heat input, and superior joint properties. However, they are constrained by harsh welding environments and high operational costs [4,5,6]. Traditional tungsten inert gas (TIG) welding is widely adopted for its flexibility and ability to weld complex structural components. Nevertheless, it necessitates wide bevels and employs multi-layer, multi-pass welding techniques for thick titanium alloy plates, leading to prolonged welding times and increased costs [7]. Narrow-gap TIG welding, by reducing the welding current and controlling arc oscillation, enables high-quality welded joints with small bevels, making it the preferred method for welding large, complex titanium alloy components [8,9,10,11]. To control heat input and ensure full sidewall penetration, narrow-gap TIG welding uses slow welding speeds and small single-pass weld layers, still resulting in inefficiencies [9]. Additionally, at low currents, conventional TIG arcs are prone to instability from external disturbances, necessitating precise control of the auxiliary arc oscillation device [10,11].

K-TIG has emerged as an effective and high-quality welding technique for medium–thick plates (3–30 mm) and is widely utilized in the metal industry [12,13,14,15,16,17,18,19]. K-TIG employs a specialized water-cooled torch that allows high currents (≤600 A) to create a compressed arc, resulting in a small hole that enables single-side welding with double-side formation for Ti-6Al-4V titanium alloy plates up to 12 mm thick, eliminating the need for bevels and filler metals. The resulting welded joints feature fine grains and excellent performance, making it an ideal method for root welding [13]. Recent research indicates that, compared to conventional TIG welding, K-TIG welding achieves a significant cathode focusing effect at currents between 200 and 350 A, which enhances arc penetration depth and reduces the molten pool diameter [14,15]. Liu [16,17] found that the K-TIG arc consists of a stable internal compressed arc and an external free arc. The internal arc generates high energy density in the cathode region, promoting fusion, while the external arc expands the molten pool, optimizing weld formation. These features make K-TIG welding particularly suitable for efficiently filling thick plates. Moreover, the high arc stiffness ensures stability during oscillating filling [15,16].

Building upon the literature review mentioned above, this study introduces a Mechanical Arc Oscillation K-TIG hot-wire welding technology. This novel approach addresses the challenge of balancing high welding efficiency with low costs in the welding of titanium alloy thick plates. To meet the toughness requirements of marine engineering applications, Ti-6Al-4V titanium alloy with the Widmanstätten microstructure is used as the base material (BM) for welding. The microstructure and mechanical properties of the welded joints at various locations are systematically characterized and analyzed. This study aims to provide a foundation for the development of low-cost, high-efficiency welding technologies for titanium alloy thick plates, contributing both conceptual insights and theoretical knowledge.

## 2. Experimental Materials and Methods

### 2.1. Experimental Materials

**Figure 1:**
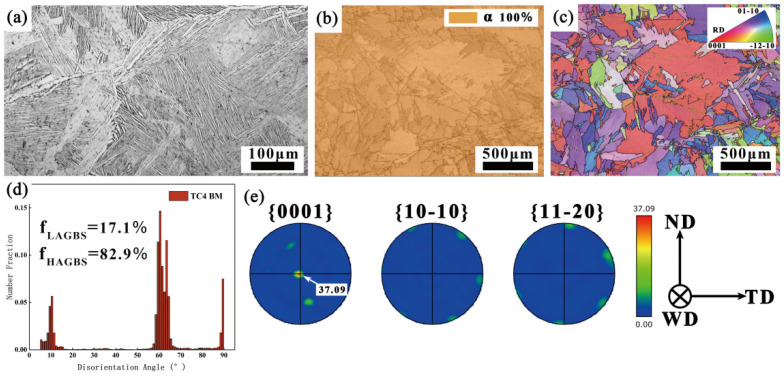
OM and EBSD analysis of BM: (**a**) OM; (**b**) PM; (**c**) IPF; (**d**) MAD; (**e**) PF.

The BM used in this study is Ti-6Al-4V, supplied by Shaanxi Baoji Titanium Industry Co., Ltd. (Baoji, China). The material was prepared through β-phase annealing. The dimensions of the specimen are 323 mm × 180 mm × 95 mm. Figure 1 illustrates the optical microscopy (OM) and electron backscatter diffraction (EBSD) images of the BM. As shown in Figure 1a, the BM exhibits a typical Widmanstätten microstructure, where the α phase grows along the prior-β grain boundaries and the α colony inward from the grain boundaries within the prior-β grains. The α phases within the same cluster are parallel to each other and share a common crystallographic orientation, with a small β-phase intermediate layer separating them. The phase distribution map of the BM, obtained through EBSD (Figure 1b), shows that the material is predominantly composed of the α phase, highlighting the orientation distribution of this phase. The misorientation angle distribution (pixel-to-pixel) analysis (Figure 1d) reveals that high-angle grain boundaries (HAGBs) make up 82.9% of the BM, while low-angle grain boundaries (LAGBs) comprise only 17.1%. The inverse pole figure (IPF) maps (Figure 1c,e) indicate that the BM predominantly exhibits a <0001> crystallographic orientation, with the maximum pole density of the texture strength reaching 37.09. The nominal composition of the filler metal used is Ti-6Al-3V, with a diameter of 1.2 mm. The detailed chemical composition of both the base material and the filler metals is provided in Table 1, and the mechanical property data can be found in Table 2.

### 2.2. Welding Method

**Figure 2:**
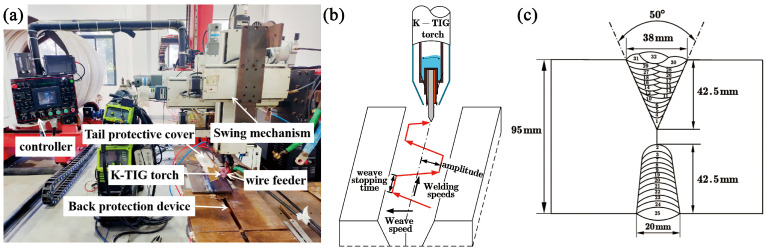
Welding platform and method: (**a**) welding system; (**b**) welding torch swing circuit diagram; (**c**) welding groove and bead distribution.

This study utilizes the K-TIG hot-wire welding technique combined with mechanical arc oscillation to perform double-sided alternating multi-layer, multi-pass welding on titanium alloy thick plates. The groove design of the workpiece and the alternating welding passes are shown in Figure 2c. A total of 32 welding passes were completed in the experiment, with the interlayer temperature controlled at approximately 60 °C by a DT1311-type contact thermometer, produced by Shenzhen Lihuajin Technology Co., Ltd., Shenzhen, China. The root layer has a thickness of 17 mm, and the average thickness of each filling layer is 2.5 mm, significantly improving welding efficiency.

The welding platform is shown in Figure 2a,b, where the workpiece is securely fixed to the platform with bolts, and the welding torch is integrated with the rear protective cover. During the welding process, the workpiece is fully shielded by the rear protective cover and the backside ventilation shield. Argon gas, with a flow rate of 25 L/min, is used as the shielding gas. The height of the tungsten electrode is manually adjusted to control the arc length, while the transverse oscillation and forward speed of the welding torch are precisely controlled by the system, ensuring the automation of the welding process. The dwell time of the welding torch during oscillation is set to 0.3 s, and the hot-wire current is maintained at 180 A. Additional welding parameters are provided in Table 3.

### 2.3. Microstructure and Performance Testing Method

**Figure 3:**
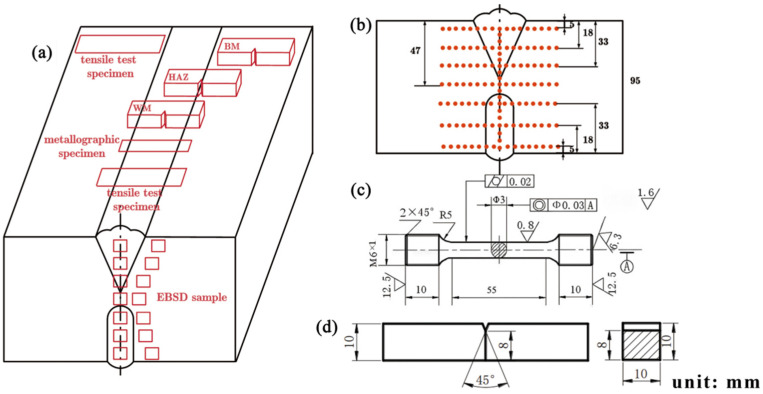
Sampling scheme of specimens: (**a**) sampling location distribution; (**b**) microhardness testing distributions; (**c**) tensile specimen; (**d**) impact specimen.

After completing the process experiments, specimens were taken from the workpiece for mechanical performance testing and microstructure analysis, as illustrated in Figure 3. Metallographic specimens were prepared through cutting, grinding, and polishing, followed by etching using a mixed solution of 1 vol-% HF, 3 vol-% HNO_3_, and 7 vol-% H_2_O. The macroscopic structure of the welded joint was observed using a VHX-7000 ultra-depth-of-field metallographic microscope, produced by Keyence Corporation, Osaka, Japan. EBSD specimens were prepared through grinding and vibratory polishing and were observed with an Oxford NOVA NANOSEM 430, produced by FEI, Hillsboro, OR, USA, with analysis performed using AZtecCrystal 2.12 software. Vickers hardness testing was carried out on various regions of the welded joint using a microhardness tester (HVS-1000Z, produced by Shanghai Yizong Precision Instrument Co., Ltd., Shanghai, China), applying a load of 5 N for a testing time of 10 s. The interval between measurements was set to 1 mm, with specific testing locations shown in Figure 3b. Room-temperature uniaxial tensile tests were conducted on the weld metal (WM) and BM specimens using a universal testing machine (CMT 5105, produced by MTS Systems Corporation, Eden Prairie, MN, USA). The speed of the test was 3 mm/min and the initial strain rate of the test was 0.002/s. The processing dimensions of the tensile specimens are shown in Figure 3c, with each test repeated three times at each location. Room-temperature impact tests for the different regions (BM, HAZ, and WM) were carried out at ambient temperature using a PIT752H instrumented Charpy impact testing machine (Shenzhen Wan Testing Equipment Co., Ltd., Shenzhen, China), which has a 750 J capacity. A V-shaped notch was used, and its orientation is shown in Figure 3a, with the processing dimensions provided in Figure 3d. Each impact test was repeated three times at each location. After the tests, scanning electron microscopy (SEM) was employed to examine the fracture morphology of the welded joint. The equipment model is JSM-IT800, produced by JEOL Ltd., Tokyo, Japan.

## 3. Results and Discussion

### 3.1. Microstructure Morphology of the Welded Joint

#### 3.1.1. Macroscopic Morphology

**Figure 4:**
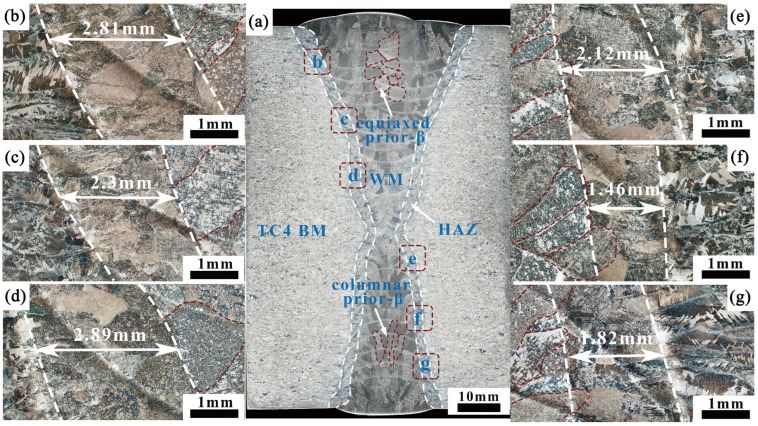
Macromorphology of the welded joint: (**a**) welded joint; (**b**) HAZ of the V-shaped top; (**c**) HAZ of the V-shaped middle; (**d**) HAZ of the V-shaped bottom; (**e**) HAZ of the U-shaped bottom; (**f**) HAZ of the U-shaped middle; (**g**) HAZ of the U-shaped top.

Figure 4a shows the macroscopic morphological characteristics of the welded joint. The Mechanical Arc Oscillation K-TIG hot-wire welding enables the formation of well-defined welded joints. The welded joint is composed of three main regions: the WM, HAZ, and BM. No significant welding defects, such as incomplete fusion or porosity, are observed.

The WM is primarily composed of columnar prior-β grains. The melting mode of the root layer follows a keyhole pattern, with the columnar grains growing transversely to the fusion line boundary. On both sides, the melting mode of the filling layers follows a conduction mode, with grains growing outward from the BM toward the maximum temperature gradient direction. Notably, the columnar grain size in the V-shaped filling layers is significantly larger than in the U-shaped filling layers. In the middle of the V-shaped filling layers, there is a certain volume fraction of equiaxed prior-β grains. The transition from columnar to equiaxed grains occurs in the overlapping zone of the multi-pass single-layer weld.

As shown in Figure 4b–f, the width and grain size of the HAZ at different positions exhibit the relationship W_bottom_ > W_top_ > W_middle_ and β_top_ < β_middle_ < β_bottom_. The HAZ width and grain size in the V-shaped filling layers are significantly larger than those in the U-shaped filling layers. In the HAZ, the grains retain the same morphology as those in the BM. The columnar grains near the fusion line grow along the direction of the maximum temperature gradient. This phenomenon is attributed to the high distribution coefficients of aluminum and vanadium in the alloy, making it difficult for these elements to nucleate before solidification [20]. The fusion line of the welded joint is clearly defined, with no significant coarse grains present in the HAZ. The width of the HAZ ranges from approximately 1.46 mm to 2.89 mm. This is because, under oscillation conditions, the welding speed increases, the net heat input is reduced, and the cooling rate is enhanced [21].

#### 3.1.2. Microscopic Morphology

**Figure 5:**
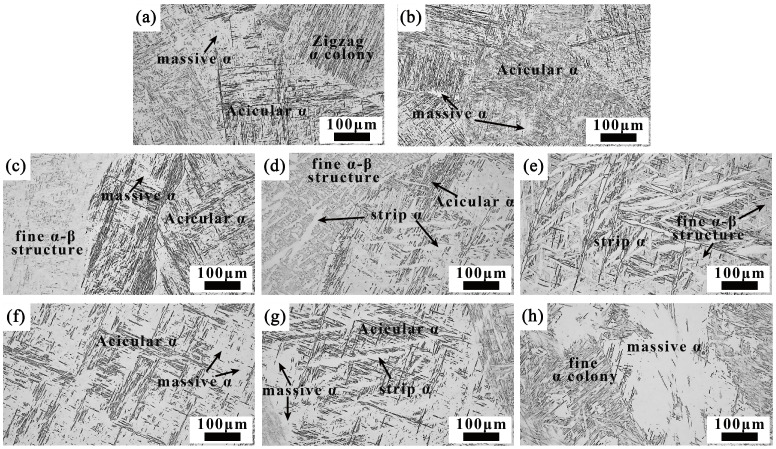
Microstructure of the welded joint at different positions: (**a**) WM of the root layer; (**b**) HAZ; (**c**) WM of the V-shaped bottom; (**d**) WM of the V-shaped middle; (**e**) WM of the V-shaped top; (**f**) WM of the U-shaped bottom; (**g**) WM of the U-shaped middle; (**h**) WM of the U-shaped top.

Figure 5 illustrates the microstructure of the welded joint across different regions. As shown in Figure 5a, the K-TIG welded joint does not exhibit the α’ martensitic phase due to the high heat input (2890 kJ/m). Instead, it consists of numerous interwoven acicular α phases, a zigzag α colony, and massive α phases [13,18]. Unlike steel, titanium alloys do not experience significant recrystallization upon cooling after being heated beyond the phase transition temperature. Since the BM initially has an elongated Widmanstätten structure, the HAZ of the welded joint retains the prior-β grain morphology. During cooling, the internal α colony undergoes a phase transformation into interwoven acicular α phases, along with a small amount of massive α phases (Figure 5b).

Under high-current conditions, multi-layer multi-pass TIG welding undergoes complex thermal cycles, resulting in a highly variable microstructure [22]. Previous studies on the cooling rate and heat input during TIG welding of titanium alloys indicate that the heat input during the filling welding stage of this experiment ranged from 1202.7 to 1420.36 kJ/m, with corresponding cooling rates of 36 to 110 °C/s [7]. After each welding pass, the weld primarily undergoes a diffusion phase transformation, with the microstructure dominated by massive α and various forms of α phases. In certain regions, acicular α’ martensite can be found [7,23]. Furthermore, the multi-layer multi-pass filling welding provides a form of post-weld heat treatment (PWHT), which promotes the decomposition of acicular α’ and some massive α phases into fine α+β structures [22,24]. Consequently, the welded joint microstructure is predominantly composed of different forms of α phases formed during cooling, as well as fine α+β structures generated during PWHT (Figure 5c–h).

The filling methods on either side of the welded joint groove vary, leading to significant differences in the microstructure. In the case of a V-shaped groove, a multi-layer multi-pass filling method is employed, resulting in higher heat input and more uniform heat dissipation, which increases the size of the α phases. In this case, the strip α phases are relatively short, wide, and interwoven with each other (Figure 5c–e). In contrast, for a U-shaped groove, a multi-layer single-pass filling method is used. As the number of filling layers increases, the swing amplitude of the scanning arc heat source enlarges, causing the strip α phases in the middle of the filling layers to grow in the direction of the swing, resulting in a long strip morphology. Due to the absence of subsequent weld passes for PWHT, the microstructure at the top of the filling layers differs from that of other regions. It is primarily composed of large areas of massive α phases and a fine α colony along the interfaces of the massive α phases (Figure 5f–h).

### 3.2. Grain Orientation and Distribution Analysis

**Figure 6:**
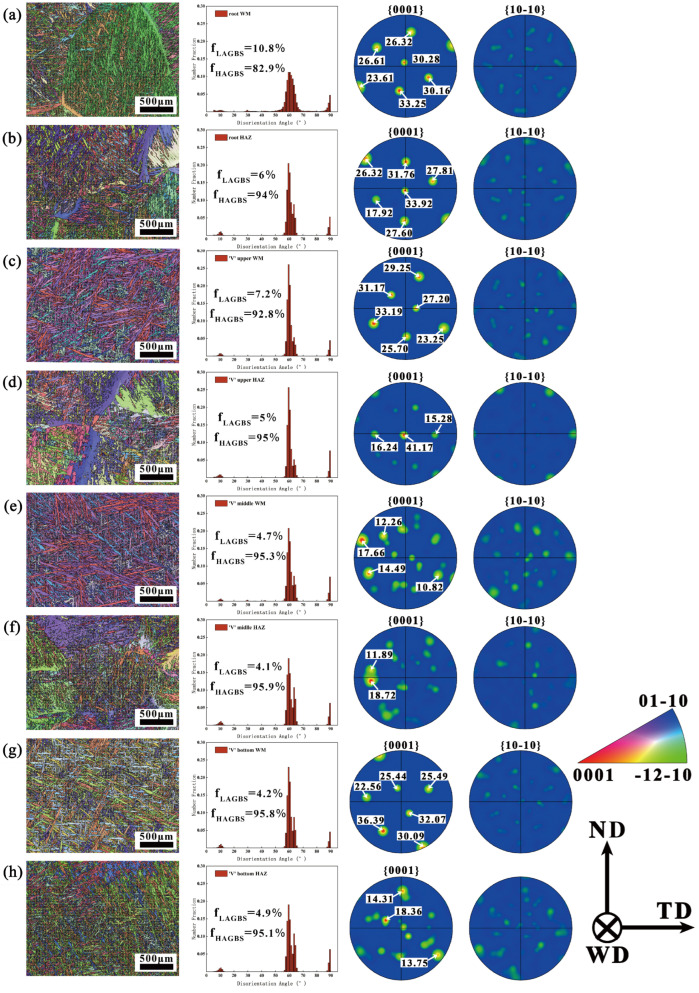
EBSD analysis of V-shaped and root layers: (**a**) WM of the root layer; (**b**) HAZ of the root layer; (**c**) WM of the V-shaped top; (**d**) HAZ of the V-shaped top; (**e**) WM of the V-shaped middle; (**f**) HAZ of the V-shaped middle; (**g**) WM of the V-shaped bottom; (**h**) HAZ of the V-shaped bottom.

Figure 6 displays the EBSD analysis of the root and V-shaped filling layers. Comparing the orientation difference distribution (pixel-to-pixel) statistics of the BM with the WM and HAZ of the root and filling layers reveals a significant increase in the proportion of HAGBs. The grain boundary angles are primarily concentrated around 10°, 60°, and 90°. The WM in the root layer exhibits a higher proportion of LAGBs compared to the filling layers, which can be attributed to dislocations generated by rapid heat dissipation during keyhole mode welding [25]. Since the BM, HAZ, and WM of the Ti-6Al-4V titanium alloy plate used in this study are almost entirely composed of the α phase, the analysis focuses on the texture types and intensity variations of the α phase.

The PF at different positions in Figure 6 show that the highest texture strength at all positions corresponds to the basal plane {0001}. The texture of the WM in the root layer is nearly identical to that of the BM, with both exhibiting a {0001}<0001> plate texture. This similarity is attributed to the welding direction following the RD direction and the use of the keyhole mode. Research by Cui, Ou, and others confirmed that under single-side welding with double-side forming conditions in K-TIG welding, the texture of the WM resembles that of the BM [13,26]. During the filling welding stage, which uses a scanned arc heat source, the texture types and strengths of the WM and HAZ change significantly. The IPF map in Figure 6 illustrates that, under the influence of the scanned arc, different types of α phases interlace, creating a variety of colors, which increases the texture types in the WM and HAZ.

Except for the WM in the root layer, the texture strength in the WM and HAZ at other locations is relatively weaker compared to that in the BM, with the HAZ exhibiting a notably weaker texture than the WM. This indicates that the multi-cycle thermal effects of welding effectively increase the number of texture types in the HAZ while weakening the texture strength. This finding contrasts with observations from conventional low-current TIG multi-layer multi-pass welding, where the multi-cycle thermal effects result in the HAZ and WM displaying higher texture strengths than the BM [25]. The discrepancy may be attributed to the extended high-temperature exposure time in the HAZ during high-current repeated filling, which leads to recrystallization, grain growth, and other structural changes.

**Figure 7:**
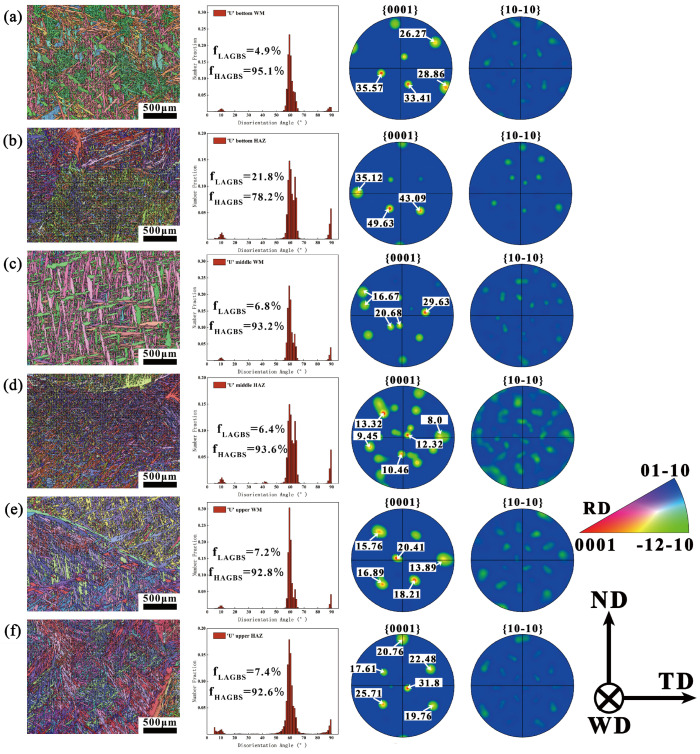
EBSD analysis of U-shaped layers: (**a**) WM of the U-shaped bottom; (**b**) HAZ of the U-shaped bottom; (**c**) WM of the U-shaped middle; (**d**) HAZ of the U-shaped middle; (**e**) WM of the U-shaped top; (**f**) HAZ of the U-shaped top.

Figure 7 presents the EBSD analysis of the U-shaped filling layers. The analysis results indicate that the LAGBs in the HAZ at the top of the U-shaped filling layers are significantly higher than those at other locations. This is likely due to fewer thermal cycles at this location, coupled with faster heat dissipation near the surface, which promotes the formation of a large number of dislocations. As a result, there is a significant reduction in HAGBs in this region [25]. The texture evolution in the U-shaped filling layers follows a pattern similar to that in the V-shaped filling layers, although the texture strength in the U-shaped filling layers is relatively lower. Considering the differences in filling methods and the original columnar grain sizes across the various filling layers, this study suggests that the texture strength is closely related to the formation of coarse columnar grains in the weld bead and the multiple cyclic thermal effects occurring during the welding process [27,28]. Moreover, the extent of the impact of these cyclic thermal effects varies under different welding currents.

### 3.3. Mechanical Properties of the Welded Joint

#### 3.3.1. Microhardness

**Figure 8:**
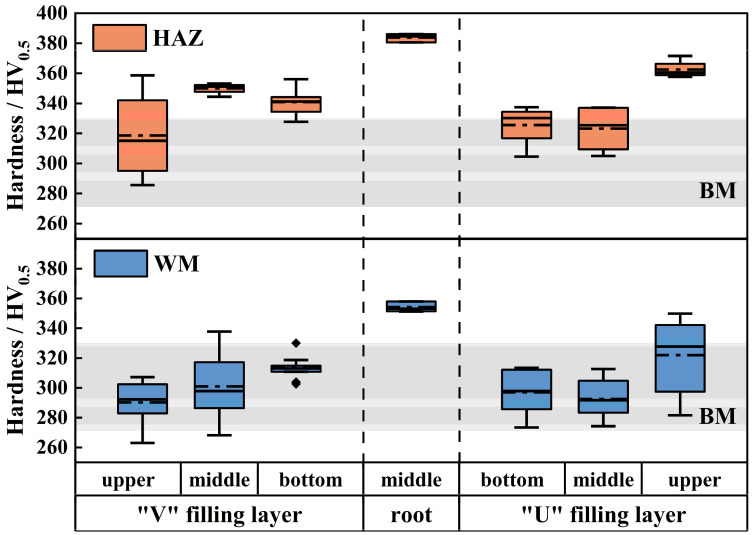
Microhardness diagram of the welded joint.

Figure 8 presents the hardness distribution of the welded joint. The results reveal that the hardness of the root layer’s WM and its HAZ is significantly higher than that of the BM, while the hardness of the filling layers on both sides is comparable to that of the BM, with a slight increase in the HAZ hardness. Specifically, the hardness of the BM ranges from 271 to 330 HV_5_, the hardness of the root layer ranges from 351.3 to 386.1 HV_5_, the hardness of the filling layers ranges from 263 to 337 HV_5_, and the hardness of the HAZ ranges from 285 to 371.6 HV_5_.

The significant difference in hardness between the filling and root layers is likely due to the inherently lower hardness of the filling metal. Furthermore, the large variation in the hardness distribution of the upper filling layer at the U-shaped groove, as well as the marked increase in HAZ hardness, may be linked to the larger oscillation amplitude in this region. The absence of PWHT from subsequent weld passes also contributes to this phenomenon. The increased oscillation speed accelerates the cooling rate of the weld bead, causing significant differences in the thermal cycles experienced by different areas of the welded joint.

#### 3.3.2. Tensile Property

**Figure 9:**
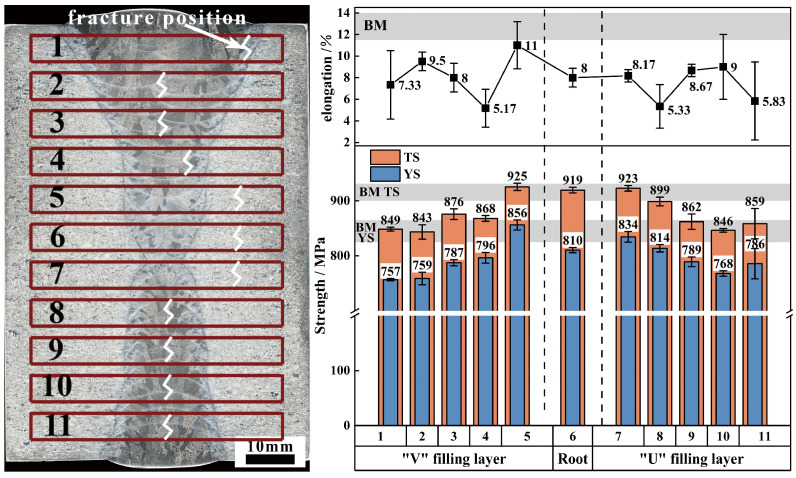
Comparison of tensile properties of the welded joint.

Figure 9 presents a comparative analysis of the tensile property of the welded joint. It is evident that the strength of the welded joint is slightly lower than that of the BM, with variations in plasticity across different regions. Specifically, the root layer and its adjacent filling layers exhibit the highest strength, comparable to that of the BM, with fractures occurring in the BM. The strength and plasticity trends of the filling layers on both sides are similar, indicating that the tensile property of the filling layers are minimally affected by the groove form. The tensile strength of the filling layers ranges from 843 to 868 MPa, which represents over 92.6% of the BM’s tensile strength (910–927 MPa), while the yield strength ranges from 757 to 814 MPa, over 91.7% of the BM’s yield strength (825–865 MPa).

Regarding plasticity, the weakest regions of the filling layers are primarily located at the bottom and top (#1, #4, #8, #11), with an elongation rate approximately 40% that of the BM. However, the middle section of the filling layers demonstrates good plasticity, with a post-fracture elongation rate of 8–9.5%, reaching up to 70% of the BM’s value. Nevertheless, fracture locations differ between the two sides. Fractures at the V-shaped grooves occur in the HAZ near the BM or the interpass HAZ of the weld beads, while fractures at the U-shaped grooves occur at the center of the WM. Further examination of the tensile fracture morphology reveals nearly identical fracture surfaces for the filling layers at the same position on both sides. A typical tensile fracture is selected for further analysis.

**Figure 10:**
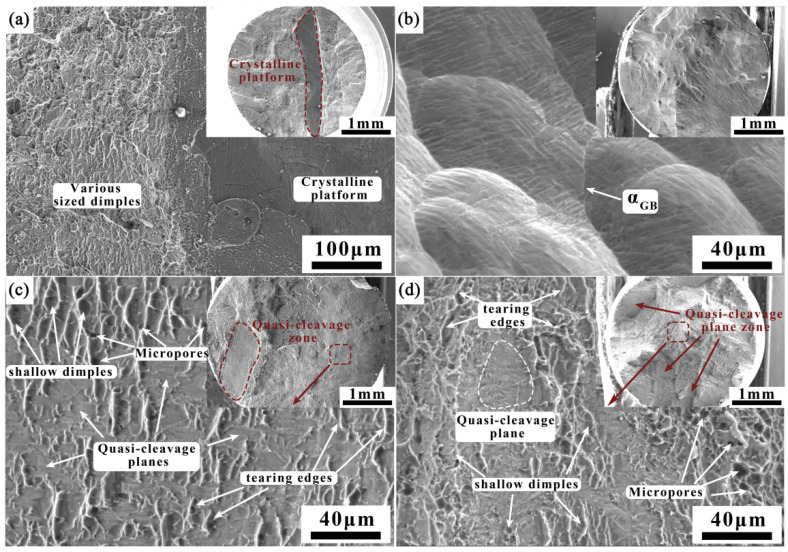
Fracture morphology of weak parts of the welded joint: (**a**) #1; (**b**) #11; (**c**) #5; (**d**) #8.

Figure 10 illustrates the fracture morphology of the weak regions in the welded joint. Fracture #1 displays a distinct crystalline platform region (Figure 10a), while Fracture #11 covers a large area of “microstructure” (Figure 10b). Under high magnification, the prior-β grains and boundary α phase within the fracture are clearly identifiable. These observations indicate regions at the top of the filling layers on both sides where bonding strength is insufficient, leading to poorer plasticity in these areas. The fracture morphology of Fractures #5 and #8 is highly consistent, with a visible quasi-cleavage zone on a macroscopic level, while other areas are characterized by smaller quasi-cleavage planes, shallow dimples, and tearing edges (Figure 10c,d). Micropores are also observed within the fracture. The transverse residual stress within the welding manifests as tensile stress, significantly predisposing the joint to fracture during transverse tensile loading. In the context of thick-plate, multi-layer, multi-pass welding, the peak residual welding stress consistently localizes at the 25% plate thickness position [29], corresponding precisely to measurement locations #4 and #8 on the specimen investigated in the present study. To confirm that residual stress is the primary cause of reduced plasticity, 700 °C/1 h + air cooling de-stress annealing was applied to these positions, followed by tensile tests. The results are presented in Table 4. As shown in the Table 4, after de-stress annealing, the welded joint’s strength remains largely unchanged, but its plasticity significantly improves. Furthermore, the fracture locations of the samples remain consistent after annealing.

**Figure 11:**
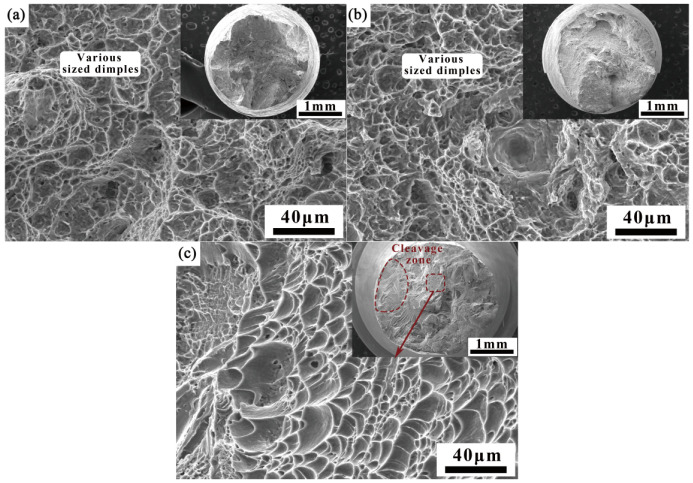
Fracture morphology of other parts of the welded joint: (**a**) #2; (**b**) #10; (**c**) #6.

Figure 11 depicts the fracture morphology of other regions of the welded joint. As shown in Figure 11a,b, the central fracture of the filling layers on both sides exhibits a typical ductile fracture, with deep and shallow ductile dimples of varying sizes. It can be seen from Figure 11c that the root layer and fractures near the filler layers exhibit a mixed fracture mode, with severe plastic deformation observed on the cross-section, showing distinct cleavage zones and deep ductile dimple regions.

#### 3.3.3. Impact Property

**Figure 12:**
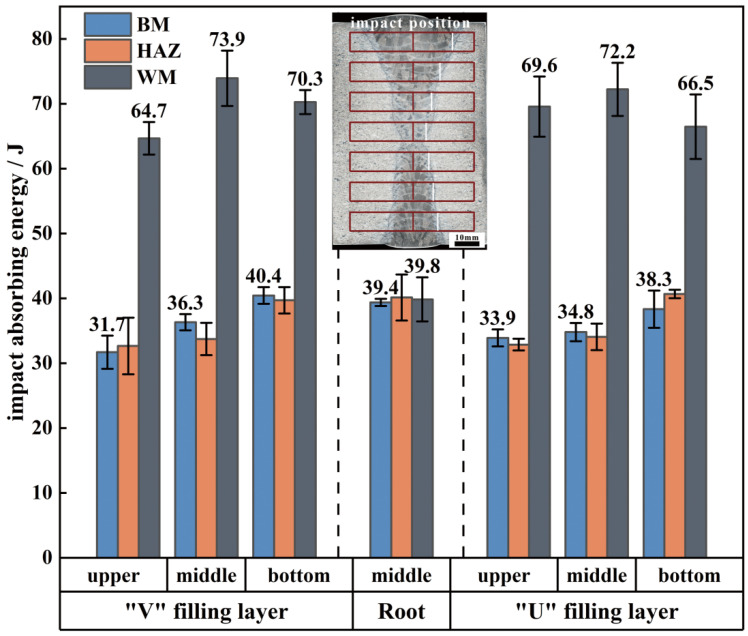
Comparison of impact properties of the welded joint.

**Figure 13:**
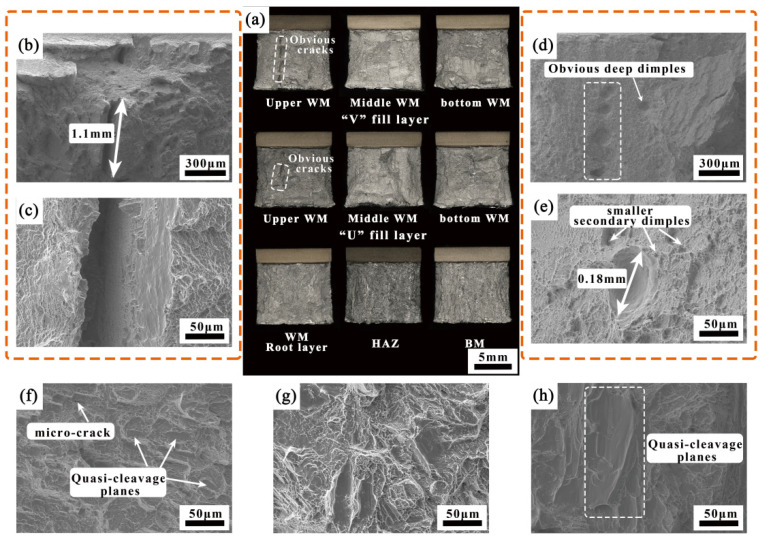
Impact fracture morphology of the welded joint.

Figure 12 presents a comparison of the impact toughness of the welded joint. As observed, although the strength and plasticity of the welded joint are lower than those of the BM, its impact performance is exceptional. The impact absorption energy of all layers’ HAZ and the root layer’s WM is approximately equal to that of the BM, while the impact absorption energy of the filling layers ranges from 64.7 to 73.9 J, nearly double that of the BM. Figure 13 illustrates a comparison of the impact fracture surfaces of the welded joint. As shown in Figure 13a, the macro-fracture of the WM exhibits more pronounced plastic deformation compared to those of the HAZ and the BM. At the top of the filling layers, long, tear-shaped cracks are clearly visible, with lengths reaching up to 1.1 mm (Figure 13b). A closer examination of the crack region reveals numerous ductile dimples and tearing edges, indicating that pre-existing plastic deformation occurred in this area, characteristic of a ductile fracture mode (Figure 13c). The fracture morphology of the filling layers in the middle and bottom regions is largely consistent, with a high density of deep, sizable ductile dimples. The diameter of these dimples is approximately 0.18 mm, with smaller secondary dimples densely distributed around the larger ones, forming a typical small-dimple fibrous fracture (Figure 13d,e) and contributing to the superior impact property in this area. As shown in Figure 13f–h, the fracture surfaces of the root layer’s WM, HAZ, and BM primarily consist of quasi-cleavage planes, dense shearing ridges, and ductile dimples, with microcracks present in some areas, indicating a mixed fracture mode.

In recent years, titanium alloy TIG welded joints have demonstrated superior impact toughness compared to the BM. This phenomenon has been validated by numerous studies, with the academic community generally agreeing that the tortuosity of the crack propagation path is a key factor in enhancing the impact toughness of titanium alloy welded joints. However, there is no consensus on the primary factors influencing the tortuosity of these crack propagation paths. Cui [13] proposes that an increased proportion of high-angle grain boundaries in the WM is a significant factor contributing to improved impact toughness in welded joints. Zhou [30] contends that microcracks tend to form at the grain boundaries of the primary α phase, which are challenging to impede. They attribute the observed increase in impact toughness to the reduced primary α-phase content in TIG welding. Gao [31] suggests a positive correlation between microstructural orientation and impact toughness, highlighting that the significant orientation differences generated during welding can enhance the impact toughness of titanium alloys. S. Lathabai [19] asserts that the high aspect ratio of the α phase in the WM provides a tortuous interface for crack propagation, thereby complicating the crack path and improving the impact toughness. In this study, the BM exhibits a Widmanstätten structure consisting of various α clusters, and both the WM and the HAZ display similar α-phase sizes, characterized by a high proportion of large-angle grain boundaries and a notable distribution of orientation differences.

Based on this theoretical framework, it was anticipated that the impact energy absorption in the welded joint would follow this trend: WM ≈ HAZ > BM. However, experimental results indicate that impact energy absorption in the WM is significantly higher than in the other regions, while the impact energy in the HAZ is comparable to that in the BM. Therefore, the existing theoretical models fail to fully account for the unusually high impact toughness observed in the WM in this study.

Compared to other welding methods, TIG welding results in higher heat input, which leads to increased residual stress within the welded component. Additionally, the transverse mechanical oscillation of the arc amplifies this residual stress. During thick-plate filling welding, excessive residual stress, combined with varying cooling rates at different locations of the welded joint, results in the tight interlocking of different α-phase morphologies, which enhances the engagement strengthening effect [29]. This microstructure hinders crack initiation and propagation under impact loading conditions. As a result, the fracture surface of the impact-tested specimens from the filling layers exhibits significant fluctuations, displaying typical ductile fracture characteristics. In the ductile fracture mode, the material undergoes substantial plastic deformation, absorbing more energy and consequently showing superior impact toughness [32]. In contrast, the root layer employs keyhole welding technology, which ensures uniform heat dissipation and enables full release of welding stress, without noticeable engagement strengthening. Therefore, the impact energy absorption of the root layer is considerably lower than that of the filling layers.

Unlike the fracture mechanisms in tensile specimens, where primary voids aggregate through secondary voids, leading to similar fracture types, the Charpy “V” notch impact test reveals two distinct fracture types: coalescence fracture caused by the growth of defects, such as voids and cracks, and small-dimple fibrous fracture [33]. Previous experimental results have identified regions at the top of the filling layers with weaker bonding strength, which contain a higher concentration of defects. Under the influence of impact force, adjacent defects overcome surrounding obstacles, leading to coalescence and expansion until fracture occurs. The shrinkage strain induced during welding, along with the heat treatment effect of the upper weld metal on the lower weld metal, results in a complex internal structure within the weld metal, significantly hindering crack propagation in the specimen. Consequently, despite the lower strength and plasticity at the top of the filling layers, its impact toughness is considerably higher than that of the BM.

## 4. Conclusions

This study experimentally verifies the effectiveness of the mechanical arc oscillation K-TIG hot-wire filling welding technology for welding 95 mm-thick Ti-6Al-4V titanium alloy plates. In this approach, the root-layer thickness can reach up to 17 mm, while the average thickness of the filling layers per pass is 2.5 mm. Compared to conventional TIG welding, this method significantly enhances welding efficiency. It employs high-current filling to increase cladding efficiency, while the mechanical oscillation of the high-speed arc effectively controls the heat input per unit during the welding process. Consequently, the HAZ width of the welded joint is reduced, and the prior-β-phase grain size in the weld does not exhibit a significant increase as expected. Although the cover layer of the welded joint shows weak bonding, further optimization of the process can fully improve performance. Based on the microstructure and property analysis of the welded joint, and a comparison with relevant literature, the following conclusions can be drawn:

1. The mechanical arc oscillation K-TIG hot-wire filling welding technology is successfully applied to weld 95 mm-thick Ti-6Al-4V alloy workpieces, demonstrating high welding efficiency and good joint properties.
2. Under the engagement strengthening effect, the strength of the welded joint can reach more than 92.6% and the elongation is 40~70% of the BM. The hardness of the root weld is approximately 351.3–386.1 HV5, while the hardness of the filler metal weld ranges from 263 to 337 HV_0.5_, and the hardness of the heat-affected zone lies between 285 and 371.6 HV_0.5_. The significant difference in hardness between the filler and root welds is likely due to the inherently lower hardness of the filler material.
3. The WM microstructure primarily consists of various forms of α phases and fine α+β structures. The HAZ is characterized by interlaced acicular α phase and a small amount of massive α phase. The α phase disperses in a relatively wide, multidirectional pattern.
4. The impact of multi-cycle thermal effects on texture types and strength varies with different welding currents. In high-current filling welding, multi-cycle thermal effects can effectively increase the diversity of texture types in the HAZ while weakening the texture strength.
5. The joint exhibits excellent impact property, with the impact absorption energy of the filling weld seam ranging from 64.7 to 73.9 J, approximately twice that of the BM. The impact toughness in other areas is comparable to that of the BM, with no noticeable weak spots. The contribution of the engagement strengthening effect in TIG thick-plate welding to the impact toughness of the filling layers should not be overlooked.

## Figures and Tables

**Table 1:** Compositions of the base material and filler metal.

Material	Al	V	Fe	O	C	Ti
Base material	6.08	3.82	0.14	0.18	0.034	bal.
Filling metal	5.96	2.78	0.015	0.043	0.005	bal.

**Table 2:** Mechanical properties of the base material and filler metal.

Material	UTS/MPa	YS/MPa	Elongation/%
Base material	900~931	825~865	11.5~14
Filler metal	826	677	13.8

**Table 3:** Welding process parameters.

	Current (A)	Weld Speed (mm/min)	Wire Feed Speed (mm/min)	Weave Speed (mm/min)	Amplitude (mm)
Root weld	600	200	——	——	——
Filling weld	220~320	130	2000~6000	100~130	3~6

**Table 4:** Comparison of tensile properties of weak positions under different conditions.

Sample Number	Sample State	UTS/MPa	YS/MPa	Elongation/%
#4	As-welded	868	796	5.17
	Annealing	873	799	7.5
#8	As-welded	899	814	5.33
	Annealing	901	831	6.5

## Data Availability

The original contributions presented in this study are included in the article. Further inquiries can be directed to the corresponding authors.
